# On the recognition of the long neglected *Vitisadenoclada* Hand.-Mazz. (Vitaceae) from southern China

**DOI:** 10.3897/phytokeys.179.65519

**Published:** 2021-06-17

**Authors:** Jun Wen, Zhi-Yao Ma

**Affiliations:** 1 Department of Botany, National Museum of Natural History, Smithsonian Institution, PO Box 37012, Washington, DC 20013-7012, USA National Museum of Natural History, Smithsonian Institution Washington United States of America

**Keywords:** Grapes, species delimitation, Vitaceae, *Vitis*, *Vitisadenoclada*, *Vitisheyneana*

## Abstract

This study reports the recognition of *Vitisadenoclada* Hand.-Mazz. from southern China. The species was not recognized in the *Flora Reipublicae Ropularis Sinicicae* and *Flora of China* treatments. Recent field studies and examination of herbarium collections including the type material suggest that *Vitisadenoclada* is morphologically similar to *V.heyneana*, in their densely arachnoid tomentose abaxial leaves, yet it can be easily distinguished from the latter by its red-purple glandular hairs on the young branches (vs. glandular hairs absent in *V.heyneana*) and inflorescences usually subtended by a tendril at the base (vs. only occasionally with a tendril in *V.heyneana*). *Vitisadenoclada* may be a species of hybrid origin, with the highly tomentose *Vitisheyneana* as one of the parental species, and likely the glandular-hair bearing *V.davidii* as the other parental species. *Vitisadenoclada* is recorded from southern China in Guangdong, Guangxi, Guizhou, Hunan and Zhejiang provinces.

## Introduction

*Vitis* L. (the grape genus) consists of about 75 species widely distributed in the Northern Hemisphere, with eastern Asia and North America as its current centers of diversity (Wen 2007; Wen et al. 2018a, b; Ma et al. 2020). Li (1998) and Ren and Wen (2007) did not treat *Vitisadenoclada* Hand.-Mazz., which was described from Hunan by Handel-Mazzetti (1925), in the *Flora Reipublicae Ropularis Sinicicae* and *Flora of China* treatments. Species delimitation of eastern Asian *Vitis* remains highly problematic (Li et al. 1996; Wan et al. 2008; Ma et al. 2018, 2020). Based on our recent field work and the examination of the type material and additional specimens, we herein propose to recognize the long-neglected *Vitisadenoclada* from southern China.

## Systematics

### Vitis adenoclada

Taxon classificationPlantaeVitalesVitaceae

Hand.-Mazz., Anz. Akad. Wiss. Wien, Math.-Naturwiss. Kl. 62: 145. 1925.

0DFEF407-B2FD-534C-827C-515971B18CD9

Figures 1 , 2

Vitis adenoclada Hand.-Mazz., Anz. Akad. Wiss. Wien, Math.-Naturwiss. Kl. 62: 145. 1925. Type: China. Hunan: Xinhua Xian, near mine at Hsikwangschan (Xikuangshan), Hsinhwa (Xinhua), 600 m, 13 May 1918, in fl., *H. Handel-Mazzetti 11819* (holotype: WU, WU0029734; isotype: K, K000574937).

#### Description.

Robust high climber 3–12 m, polygamo-dioecious. Young branches with arachnoid tomentum and red purple glandular hairs, glandular hairs hardened at maturity; tendrils bifurcate to sometimes trifurcate, stout. Leaves simple; stipules brown, narrowly triangular to ovate-triangular, 3.5–5 × 2–2.5 mm, membranaceous, entire; petiole 3–7 cm, arachnoid tomentose; blade oval, 7–16 × 5–12 cm, abaxially densely tomentose, adaxially pubescent, veins 7–10 pairs, base cordate to subcordate, notch obtuse, margin serrate with 20–35 teeth on each side, notch area entire, teeth fine and short, apex acute to acuminate. Inflorescence a thyrse, leaf-opposed, 7–18 cm, paniculate in shape, usually subtended by an unbranched tendril at the base; peduncle 2–7 cm, arachnoid tomentose. Pedicel 1–3 mm, pubescent. Calyx 0.1–0.2 × 0.2–0.3 mm. Petals calyptrate, 1.5–1.8 × 0.4–0.5 mm. Stamens 2–3 mm; anthers 0.6–0.7 mm. Ovary oval; style short. Fruits purple-black, globose, 9–12 mm in diam. Seeds obovoid, chalaza rounded, ventral infolds furrowed upward 1/3–1/4 from base.

**Figure 1.**
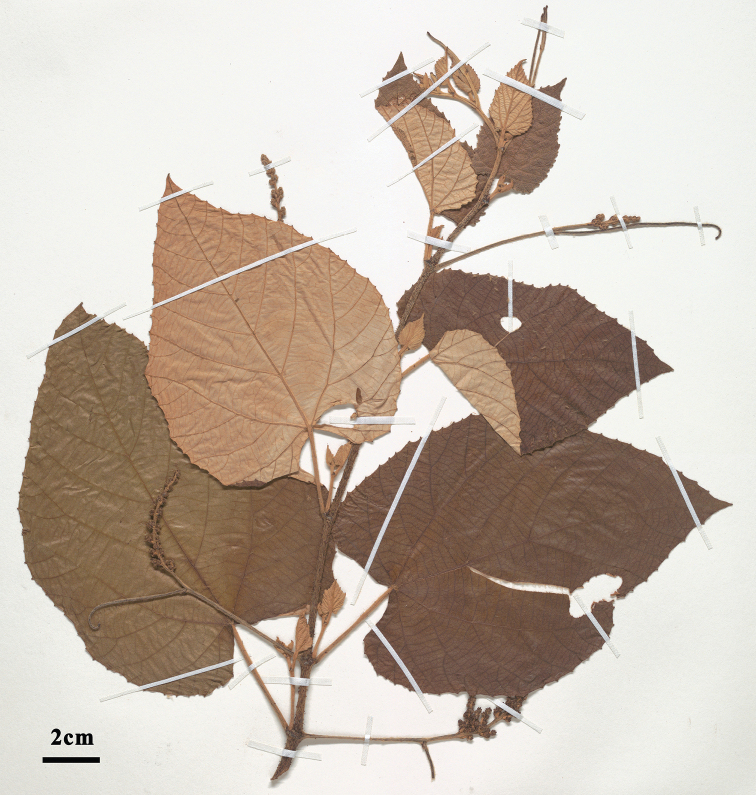
General morphology of *Vitisadenoclada*, especially showing the highly tomentose leaves and the inflorescences subtended by an unbranched tendril (*J. Wen 11560*, US).

#### Phenology.

Fl. May-Jul; fr. Jul-Oct.

#### Distribution and ecology.

In Guangdong, Guangxi, Guizhou, Hunan and Zhejiang provinces of China. 500–1015 m. Edge of forests, forests, often in limestone areas.

#### Additional specimens examined.

China. **Guangdong**: Huaiji Xian, 31 Oct 1958, *Y.G. Liu 02809* (IBSC). **Guangxi**: Jingxiu Xian, Dayaoshan, 16 km, 1015 m, 8 May 2010, floral buds, *J. Wen 11560* (US), *J. Wen 11577* (US). **Guizhou**: Jun 6, 1987, *BJFC Graduate Students 434* (BJFC00010633, BJFC00010634). **Hunan**: Hongjiang Xian, Xuefengshan, 500 m, 12 May 2014, *X.Q. Liu 114* (CCAU), *X.Q. Liu 115* (CCAU); Xinning Xian, Zhiyunshan, 13 Sep 1984, 900 m, *Zhiyunshan Exp. Team 476* (PE). **Zhejiang**: Nishui, 28 Jul 1959, fr, *S.Y. Zhang 6061* (KUN, PE).

#### Notes.

**Figure 2.**
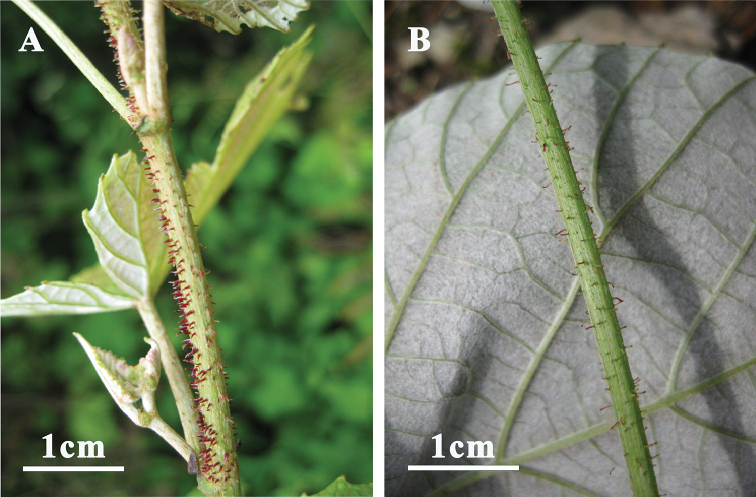
Characteristic glandular trichomes on young branches of *Vitisadenoclada***A***X.Q. Liu 114* (CCAU) **B***X.-Q. Liu 115* (CCAU) (photo credit: Z.Y. Ma).

*Vitisadenoclada* is morphologically similar to *V.heyneana* Roem. & Schult, with both having arachnoid tomentose abaxial leaves. It can be easily distinguished from the latter by its red-purple glandular hairs on the young branches (vs. glandular hairs absent on young branches in *V.heyneana*) and inflorescences usually subtended by a tendril at the base (vs. only occasionally with a tendril in *V.heyneana*) (Figures 1, 2). *Vitisadenoclada* may be a species of hybrid origin, with the highly tomentose *Vitisheyneana* as one of the parental species, and a glandular-hair bearing *Vitis* species, e.g., *V.romanetii* Rom.Caill. or *V.davidii* (Rom.Caill.) Foëx as the other potential parental species. Both *V.davidi* and *V.romanetii* share the unique glandular trichomes with *V.adenoclada* (see Ma et al. 2016), and *V.davidii* occurs in southern China in the same geographic area as *V.adenoclada*. We hence regard *Vitisdavidii* as a more likely parental species than *V.romanetii* due to their geographic distribution and the highly similar glandular trichome morphology. Ma et al. (2018, 2020) also showed that *Vitisadenoclada* and *V.davidii* grouped in one clade in the nuclear phylogeny, consistent with our hypothesis.

## Supplementary Material

XML Treatment for Vitis adenoclada
